# Left superior temporal sulcus morphometry mediates the impact of anxiety and depressive symptoms on sleep quality in healthy adults

**DOI:** 10.1093/scan/nsab012

**Published:** 2021-01-29

**Authors:** Yanlin Wang, Ping Jiang, Shi Tang, Lu Lu, Xuan Bu, Lianqing Zhang, Yingxue Gao, Hailong Li, Xinyu Hu, Song Wang, Zhiyun Jia, Neil Roberts, Xiaoqi Huang, Qiyong Gong

**Affiliations:** Huaxi MR Research Center (HMRRC), Department of Radiology, West China Hospital of Sichuan University, Chengdu, Sichuan 610041, China; Huaxi MR Research Center (HMRRC), Department of Radiology, West China Hospital of Sichuan University, Chengdu, Sichuan 610041, China; Huaxi MR Research Center (HMRRC), Department of Radiology, West China Hospital of Sichuan University, Chengdu, Sichuan 610041, China; Huaxi MR Research Center (HMRRC), Department of Radiology, West China Hospital of Sichuan University, Chengdu, Sichuan 610041, China; Huaxi MR Research Center (HMRRC), Department of Radiology, West China Hospital of Sichuan University, Chengdu, Sichuan 610041, China; Huaxi MR Research Center (HMRRC), Department of Radiology, West China Hospital of Sichuan University, Chengdu, Sichuan 610041, China; Huaxi MR Research Center (HMRRC), Department of Radiology, West China Hospital of Sichuan University, Chengdu, Sichuan 610041, China; Huaxi MR Research Center (HMRRC), Department of Radiology, West China Hospital of Sichuan University, Chengdu, Sichuan 610041, China; Huaxi MR Research Center (HMRRC), Department of Radiology, West China Hospital of Sichuan University, Chengdu, Sichuan 610041, China; Huaxi MR Research Center (HMRRC), Department of Radiology, West China Hospital of Sichuan University, Chengdu, Sichuan 610041, China; Huaxi MR Research Center (HMRRC), Department of Radiology, West China Hospital of Sichuan University, Chengdu, Sichuan 610041, China; School of Clinical Sciences, The Queen’s Medical Research Institute (QMRI), University of Edinburgh, Edinburgh EH164TJ, UK; Huaxi MR Research Center (HMRRC), Department of Radiology, West China Hospital of Sichuan University, Chengdu, Sichuan 610041, China; Research Unit of Psychoradiology, Chinese Academy of Medical Sciences, Chengdu, Sichuan 610041, China; Functional and Molecular Imaging Key Laboratory of Sichuan Province, Chengdu, Sichuan 610041, China; Huaxi MR Research Center (HMRRC), Department of Radiology, West China Hospital of Sichuan University, Chengdu, Sichuan 610041, China; Research Unit of Psychoradiology, Chinese Academy of Medical Sciences, Chengdu, Sichuan 610041, China; Functional and Molecular Imaging Key Laboratory of Sichuan Province, Chengdu, Sichuan 610041, China

**Keywords:** sleep quality, depressive symptoms, anxiety, superior temporal sulcus (STS), magnetic resonance imaging (MRI)

## Abstract

Anxiety and depressive symptoms may predispose individuals to sleep disturbance. Understanding how these emotional symptoms affect sleep quality, especially the underlying neural basis, could support the development of effective treatment. The aims of the present study were therefore to investigate potential changes in brain morphometry associated with poor sleep quality and whether this structure played a mediating role between the emotional symptoms and sleep quality. One hundred and forty-one healthy adults (69 women, mean age = 26.06 years, SD = 6.36 years) were recruited. A structural magnetic resonance imaging investigation was performed, and self-reported measures of anxiety, depressive symptoms and sleep quality were obtained for each participant. Whole-brain regression analysis revealed that worse sleep quality was associated with thinner cortex in left superior temporal sulcus (STS). Furthermore, the thickness of left STS mediated the association between the emotional symptoms and sleep quality. A subsequent commonality analysis showed that physiological component of the depressive symptoms had the greatest influence on sleep quality. In conclusion, thinner cortex in left STS may represent a neural substrate for the association between anxiety and depressive symptoms and poor sleep quality and may thus serve as a potential target for neuromodulatory treatment of sleep problems.

## Introduction

Sleep serves a critical function allowing brain restoration and repair to ensure metabolic homeostasis, clearance of neurotoxic waste products (Huang *et al.*, 2016; Fultz *et al.*, 2019) and regulation of endocrine and immune systems (Irwin *et al.*, 2003; Bonnet and Arand, 2010).

Poor sleep quality, including difficulties in initiating or maintaining sleep and sleep which is non-restorative (Montgomery and Dennis, 2004; Buysse, 2013), has been reported to be associated with abnormal emotional symptoms (Ohayon, 2002; Sateia, 2009), particularly anxiety and depressive symptoms (Breslau *et al.*, 1996; Baglioni *et al.*, 2011). Previous studies have provided evidence that anxiety and depressive symptoms may predispose individuals to sleep disturbance (Vahtera *et al.*, 2007), and sleep disturbance is an underlying feature of both anxiety and depressive symptoms (Goldstein-Piekarski *et al.*, 2020). It is possible that recurrent emotional symptoms trigger an inadequate resolution of emotional distress or biased processing of emotional information resulting in poor sleep quality (Vahtera *et al.*, 2007; Wassing *et al.*, 2019; Ben Simon *et al.*, 2020). Thus, understanding how anxiety and depressive symptoms affect sleep quality, especially the underlying neural basis, could provide an empirical basis for developing new therapeutic interventions (Cummings *et al.*, 2014; Klein *et al.*, 2018).

Recently, psychoradiologic studies have been a useful way to explore relationship between behavior and brain structure or function (Gong, 2020; Sun *et al.*, 2018; Huang *et al.*, 2019), and there is evidence that anomalies in brain function may play a role in the relationship between emotional symptoms and sleep quality. In particular, abnormal activation of medial prefrontal cortex and altered connectivity with extended limbic regions may underlie the link between anxiety and sleep disturbance (Ben Simon *et al.*, 2020) and that altered connectivity between the dorso-lateral prefrontal cortex, precuneus and lateral orbito-frontal cortex may underlie the link between depressive symptoms and poor sleep quality (Cheng *et al.*, 2018). On the other hand, studies of brain structure have shown widespread atrophy of frontal, temporal and parietal regions in people with poor sleep quality (Sexton *et al.*, 2014) and increased volume of the rostral anterior cingulate (Winkelman *et al.*, 2013), which are also distinguishing features in patients with anxiety (Pink *et al.*, 2017a) and depressive symptoms (Szymkowicz *et al.*, 2016; Pink, *et al.*, 2017b; Bartlett *et al.*, 2019). However, whether brain morphometry plays a role in the link between emotional symptoms and sleep disturbance is still unknown.

The main objective of the present study was therefore to use structural magnetic resonance imaging (MRI) and mediation analysis to identify those brain morphometric features underlying the association of emotional symptoms and poor sleep quality in a cohort of healthy adults. We hypothesized that (i) poor sleep quality was associated with cortical morphometric changes, (ii) poor sleep quality–linked cortical morphometric changes would also be associated with emotional symptoms and (iii) these cortical morphometric changes may mediate the associations between emotional symptoms and sleep quality. In addition, commonality analysis was performed to examine the unique and shared variance partitions of emotional symptoms in accounting for sleep problems to further understand the unique and common features of anxiety and depressive symptoms and their effects on sleep quality.

## Materials and methods

### Participants

Participants were recruited between August 2018 and September 2019 by means of posters and flyers distributed at West China Hospital of Sichuan University. A total of 141 healthy adults (69 women and 72 men, mean age = 26.06 years, SD = 6.36 years) who had all completed either high school (level 4), undergraduate school education (level 5) or graduate school education (level 6) were enrolled (see Table 1). Each participant completed self-reported questionnaires concerning sleep quality, anxiety and depression and MRI scanning at the Huaxi Magnetic Resonance Research Center. Exclusion criteria included a history of neurological or psychiatric disorders, sleep disorder, use of sleep medications or MRI contraindications. The study was approved by the local Research Ethics Committee, and all participants provided fully informed written informed consent.

**Table 1.** Demographic information regarding the study participants

Characteristic	Mean (s.d.)
Age (years)	26.06 (6.36)
Female, no. (%)	69 (49%)
Education level	5 (1–6)^a^
SAS	35.27 (6.63)
Affective component	7.16 (1.91)
Somatic component	21.19 (4.07)
SDS	38.78 (8)
Affective component	2.77 (0.95)
Physiological component	12.50 (2.79)
Psychological component	15.69 (3.80)
PSQI	
PSQI total score	4.71 (2.51)

^a^ Encoding for education levels: uneducated = 1, primary school = 2, junior middle school = 3, high school = 4, university = 5, post-graduation = 6.

### Behavioral measurement

The sleep quality of each participant was assessed using the Pittsburgh Sleep Quality Inventory (PSQI), which is a self-reported questionnaire relating to experience during the month prior to testing. PSQI scores range from 0 to 21, with a higher score indicating poorer sleep quality and a total score greater than 5 adjudged as indicating poor sleep quality (Buysse *et al.*, 1989).

Anxiety and depression scores for each participant were obtained using the Self-rating Anxiety Scale (SAS) and the Self-rating Depression Scale (SDS). Validation studies have demonstrated good acceptability, internal consistency and validity in the general population (Shen *et al.*, 2017). The SAS is a 20-item questionnaire which includes components relating to affective and somatic aspects of anxiety (Zung, 1971). The SDS is also a 20-item self-reporting questionnaire and includes components relating to affective, psychological and physiological aspects of depression (Zung *et al.*, 1965). For both SAS and SDS, scores in each item are added to form a total raw score with range from 20 to 80 and which was multiplied by a factor of 1.25 to obtain a so-called Index Score with a maximum value of 100. Index scores of more than 50 indicate significant anxiety and/or depressive symptoms, respectively, and higher index scores indicate more severe symptoms (Zung, 1974). A separate analysis was also performed of the separate sub-scores of SAS and SDS.

Cronbach’s α coefficient was computed using psychometric package in R-studio (https://CRAN.R-project.org/package=psychometric) as a measure of the reliability (i.e. internal consistency) of the behavioral measures. The distribution plots of the PSQI, SAS and SDS as well as the sub-scores of SAS and SDS are presented in Supplementary Figure S6.

### Image acquisition

MRI investigations were performed using a 3 T Trio imaging system (Siemens Healthineers, Erlangen, Germany). 92 participants were examined with the 32 channels phased-array head coil and 49 participants with the 12 channels phased-array head coil. For each participant, a high-spatial-resolution 3D T1-weighted spoiled gradient recalled image was acquired with repetition time 1900 ms, echo time 2.26 ms, flip angle 9°, matrix size 256 × 256, field of view 256 mm × 256 mm and slice thickness 1.0 mm. The 3D image comprises a total of 176 contiguous slices and has a voxel size of 1.0 mm × 1.0 mm × 1.0 mm. In addition, a standard diagnostic series of T2-weighted MR images were acquired using a fast spin echo sequence to check for incidental findings and to potentially exclude any participants with brain structural abnormalities from inclusion in the analysis. Earplugs and foam pads were used to protect participants’ hearing and minimize head movement during MR scanning. Images were immediately reviewed by a neuroradiologist, and repeat scanning was performed for participants for whom substantial motion or other artifacts were present in the original 3D MR image.

### Imaging preprocessing

The quality of each T1-weighted image was visually checked again to guarantee consistent data quality. The T1-weighted 3D MR images were then processed using FreeSurfer software (version 6.0.0, http://surfer.nmr.mgh.harvard.edu/) (Dale *et al.*, 1999; Fischl *et al.*, 1999), including skull stripping, alignment to Talairach space, intensity normalization, segmentation of gray matter (GM) and white matter (WM), topology correction and surface deformation along intensity gradients for optimal placement of the borders between cortical GM, WM and cerebrospinal fluid. The models of the pial surface and boundary between GM and WM were overlaid on the T1-weighted image and inspected for fidelity to visible tissue class boundaries. When inaccurate tissue delineation persisted for six or more consecutive slices, the result was deemed inaccurate (Iscan *et al.*, 2015) and correction was performed by appropriate manual editing, including addition of control points in WM voxels or removing the skull and dura when erroneously considered to be part of the brain. Next, a surface-fitting algorithm was iteratively applied to fit the GM–WM boundary in the segmented image. This surface was inflated constrained by the Laplacian of the image to generate the pial surface. The GM–WM boundary and pial surfaces were nonlinearly aligned to a symmetric surface template using a spherical registration that guarantees vertex-wise correspondence of each surface across all subjects (Fischl *et al.*, 1999).

## Statistical analyses

### Relationship between sleep quality and brain morphometry

Vertex-wise maps of cortical thickness, surface area and volume were generated for each participant using the FreeSurfer application called Qdec (query, design, estimate, contrast; http://surfer.nmr.mgh.harvard.edu). The maps were coregistered to a common spherical coordinate system and spatially smoothed with a 10 mm Gaussian kernel. A general linear model (GLM) was applied in Qdec to estimate the vertex-wise correlations of PSQI scores and the morphological parameters (i.e. cortical thickness, surface area and volume) of the left and right cerebral hemispheres separately, controlling for age, sex, education level and whether data were acquired using the 12- or 32-channel head coil. The vertex-wise maps were threshold at *P* < 0.01 and submitted to Monte Carlo null-z simulation cluster analyses with 10 000 iterations, and a cluster-level threshold of *P* < 0.01 was used to correct for multiple comparisons (Scherpiet *et al.*, 2015; Perlman *et al.*, 2017). The clusters that remained significant were used in all subsequent analyses with locations defined according to the Desikan–Killiany atlas (Desikan *et al.*, 2006). For analysis of sub-cortical (i.e. deep) GM, relevant volumes were obtained separately using the FreeSurfer automatic sub-cortical GM segmentation (Fischl *et al.*, 2002). Eight bilateral structures were studied, namely thalamus, caudate, putamen, pallidum, hippocampus, amygdala, nucleus accumbens and ventral diencephalon. The GLM tests to estimate the relationship between PSQI and the sub-cortical GM volumes controlling for age, sex, education level and head coil were performed by using SPSS software (IBM SPSS 25.0). Partial residuals of the cortical and sub-cortical GM measures showing significant associations with sleep quality after regressing out head coil covariance were calculated for use in subsequent correlation and mediation analyses.

### Correlation between anxiety and depression scores, sleep quality and brain morphometry

Partial correlation analysis with age, sex and education level as confounding variables was used to identify potentially significant relationships between anxiety and depression measures [i.e. total and sub-scores of anxiety (SAS) and depression (SDS)] and (i) the sleep quality measure (PSQI) and (ii) the brain morphology clusters significantly associated with PSQI.

### Mediation analyses

Subsequently, a mediation analysis was performed to investigate whether the sleep quality–linked brain morphometry potentially mediated a significant relationship between the independent variable (IV) being those anxiety and/or depression scores which significantly correlated with sleep quality–linked morphometry, and the dependent variable (DV) of sleep quality index (PSQI), and controlling for age, sex and education level. Mediation analysis is a path analysis used to statistically evaluate how IVs transmit their causal effects on the DV through intervening variables or mediators (Hayes, 2018; Wang *et al.*, 2020). The analysis was performed by using the Hayes PROCESS macro36 with a 10 000 bias-correction bootstrapping procedure for significance testing (Preacher and Kelley, 2011) using SPSS software (https://www.ibm.com/analytics/spss-statistics-software).

### Exploratory commonality analyses

Since 2 of the 20 items (numbers 19 and 20) in the SAS and 1 of the 20 items (number 4) in the SDS were related to the topic of sleep quality, the correlation analyses between anxiety and depression measures [i.e. total and sub-scores of anxiety (SAS) and depression (SDS)] and (i) the sleep quality measure (PSQI) and (ii) the brain morphology clusters significantly associated with PSQI, as well as the subsequent mediation analyses, were repeated with these tests eliminated to avoid any potential influence of collinearity. In addition, to obtain a better appreciation of the nature of the associations between anxiety, depressive symptoms and sleep quality, the proportions of unique and overlapping partitions among the emotional symptom sub-scores of SAS and SDS showing significant mediating effects on sleep quality were assessed using a systematic commonality analysis (Nimon *et al.*, 2008). A Venn diagram was constructed to show the relationship among these factors using the VennDiagram package in R-studio (https://www.rdocumentation.org/packages/VennDiagram) (see Supplementary Materials).

## Results

The descriptive statistics for participant demographics and sleep quality (PSQI), anxiety (SAS) and depression (SDS) measures are listed in Table 1 and plotted in Supplementary Figure S6. Cronbach’s α coefficient analysis showed good reliability for PSQI (α = 0.74), SAS (α = 0.80) and SDS (α = 0.81) confirming that the three behavior measures had a satisfactory discriminating power (Henson, 2001). Approximately 32% of participants reached the PSQI cutoff value for poor sleep quality. In addition, while most participants experienced some symptoms of anxiety and/or depression, only 3% or 6% reached the respective SDS and SAS cutoff values for mild depression and anxiety criteria.

### Relationship between sleep quality and brain morphometry

**Fig. 1.**
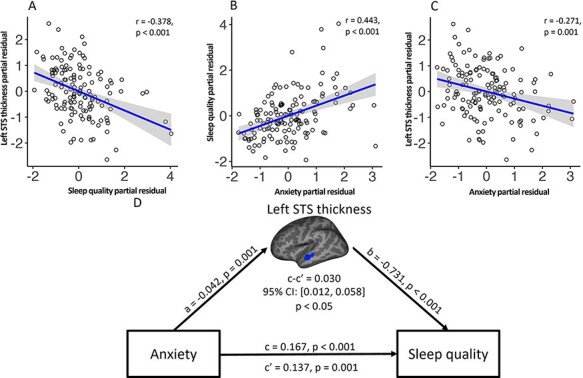
Results of mediation analysis of the association between anxiety, sleep quality (PSQI) and sleep quality–linked brain region after controlling for age, sex and education level. The upper three panels show (A) the negative correlation between cortical thickness of left STS and sleep quality, (B) the positive correlation between sleep quality and anxiety score and (C) the negative correlation between cortical thickness of left STS and anxiety score revealed by the partial correlation analysis. The lower panel (D) shows the results of the mediation analysis with a path diagram showing the strength of the associations whereby anxiety affects sleep quality through the thickness of the left STS. All data are standardized partial residuals after controlling for age, sex and education level. The indirect effect (c–c′ or a × b) was significant (for detailed information, see Supplementary Table S1).

Regression analyses were conducted to detect potential relationships between sleep quality as measured using the PSQI and brain cortical thickness, surface area and volume. A significant correlation was found between PSQI and cortical thickness for a cluster in the middle portion of left superior temporal sulcus (STS) (Monte Carlo corrected) after controlling for age, sex, education level and head coil (Table 2). In particular, higher PSQI was associated with thinner cortex in left STS (*r* = −0.378, *P* < 0.001) (Figure 1A). No other clusters were found exhibiting significant correlation between PSQI and cortical thickness, surface area or volume.

**Table 2.** Results of the association between PSQI score and surface-based brain morphometry

Cluster annotation^a^	Measure	CWP *P* value (90% CI)	Cluster size (mm^2^)	VtsMax Talairach coordinates (*x*, *y*, *z*)	Direction of effect
Left Mid-STS	Cortical thickness	[0.0077, 0.0101]	504.88	−49.0, −11.4, −12.4	Worse sleep quality, thinner

Abbreviations: Mid, middle; CI, confidence interval; CWP, cluster-wise probability; VtxMax, no. of peak vertices of the significant cluster.

^a^ Annotation of clusters according to the FreeSurfer atlas.

### Correlation between sleep quality, anxiety and depression res and brain morphometry

Partial correlation analyses with age, sex and education level as confounding variables were used to investigate the potential relationships between anxiety and depression measures [i.e. total and sub-scores of anxiety (SAS) and depression (SDS)] and (i) the sleep quality measure (PSQI) and (ii) the brain morphology clusters significantly associated with PSQI. Significant correlations were found between SAS (*r* = 0.443, *P* < 0.001) (Figure 1B), SDS (*r* = 0.407, *P* < 0.001) (Supplementary Figure S1) and PSQI scores. Similarly, significant correlations were found between SAS (*r* = −0.271, *P* = 0.001) (Figure 1C), SDS (*r* = −0.189, *P* = 0.027) (Supplementary Figure S2) and thickness of the middle portion of STS.

**Fig. 2.**
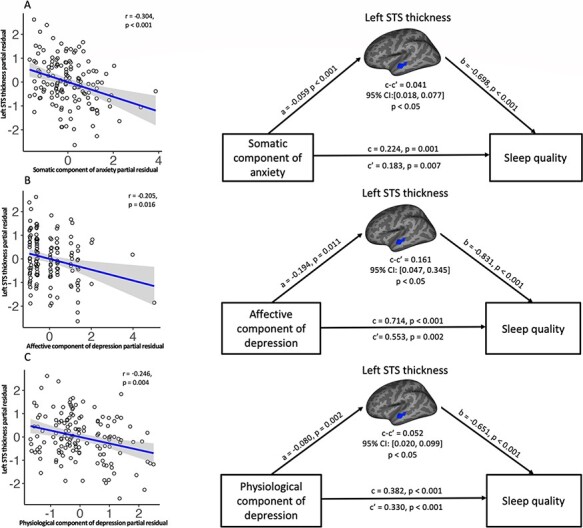
Results of mediation analysis of the association between results of SAS and SDS sub-scores, sleep quality (PSQI) and sleep quality–linked brain region, after controlling for age, sex and education level. The upper, middle and lower rows, respectively, refer to (A) the somatic component of the SAS, (B) the affective component of the SDS and (C) the physiologic component of the SDS, which all show negative correlation with cortical thickness of left STS as shown in the left panels. Path diagrams of the mediation analysis are shown in the corresponding right panels. All data are standardized partial residuals after controlling for age, sex and education level. All indirect effects (c–c′ or a × b) were significant.

For the tests for sub-scores of emotional symptoms, significant correlations were found between all the SAS sub-scores (affective component: *r* = 0.278, *P* = 0.001; somatic component: *r* = 0.477, *P* < 0.001), all SDS sub-scores (affective component: *r* = 0.340, *P* < 0.001; psychological component: *r* = 0.283, *P* = 0.001; physiological component: *r* = 0.462, *P* < 0.001) and sleep quality as measured by PSQI (see Supplementary Figure S1). In addition, significant correlations were found between all the SAS sub-scores (affective component: *r* = −0.219, *P* = 0.009; somatic component: *r* = −0.304, *P* < 0.001), two of the three SDS sub-scores (i.e. affective component: *r* = −0.205, *P* = 0.016; physiological component: *r* = −0.246, *P* = 0.004) and thickness of the middle portion of STS (see Figure 2 and Supplementary Figure S2). No significant correlation was found between the psychological component of SDS and STS thickness (*P* = 0.287).

### Mediation analysis

Mediation analysis was performed to investigate whether STS thickness could explain the association between sleep quality (DV) and anxiety and depression scores (IV), after controlling for age, sex and education level. This revealed that left STS thickness significantly mediated the effect of SAS (β = 0.030, SE = 0.012, *P* < 0.05), the somatic component of SAS (β = 0.041, SE = 0.015, *P* < 0.05), and the affective (β = 0.161, SE = 0.072, *P* < 0.05) and physiological (β = 0.052, SE = 0.020, *P* < 0.05) components of SDS on PSQI (see Figures 1D and 2, and details in Supplementary Table S1).

### Exploratory commonality analysis

**Fig. 3.**
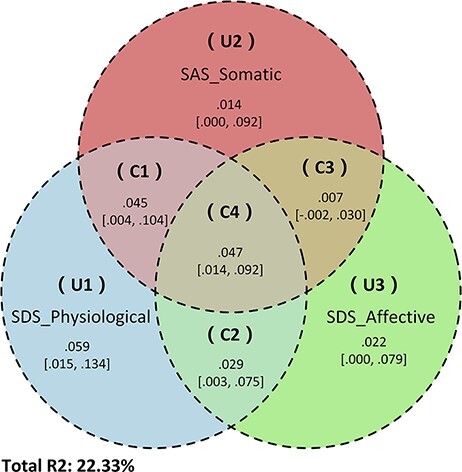
Exploratory commonality analysis using sub-scores of the somatic component of SAS, affective component and physiologic component of SDS with sleep-related items excluded as predictors and PSQI as outcomes. The upper, middle and lower rows, respectively, refer to (A) the somatic component of the SAS, (B) the physiologic component of the SDS and (C) the affective component of the SDS, which all show negative correlation with cortical thickness of left STS as shown in the left panels. Path diagrams of the mediation analysis are shown in the corresponding right panels. All data are standardized partial residuals after controlling for age, sex and education level. All indirect effects (c–c′ or a × b) were significant.

After excluding the sleep-related items in SAS (items 19 and 20) and SDS (item 4), all the significant correlations and mediation models described above remained significant (see Supplementary Figures S3–S5, Supplementary Table S2). Next, an exploratory commonality analysis was performed to obtain a better appreciation of the contributions of unique and overlapping partitions among the sub-scores of SAS and SDS which showed significant mediating effects through left STS thickness on sleep quality, namely the somatic component of SAS and the affective and physiological components of SDS, with the sleep-related items excluded from both. Overall, the combination of the three abovementioned SAS and SDS sub-scores accounted for 22.33% of the variance in PSQI. The physiological component of SDS alone accounted for 18.01% of the variance, and the combination of the somatic component of SAS and physiological component of SDS accounted for 4.5% of the variance. The physiological component of SDS accounted for an additional 5.91% of the variation in PSQI. In contrast, the somatic component of SAS, affective component of SDS and the combination of the latter two only accounted for 1.4%, 2.22% and 0.7% of the variation in PSQI, respectively. These findings are illustrated in Figure 3 and summarized in Supplementary Table S3.

## Discussion

In the current study, the brain structural variation underlying sleep quality and the association of sleep quality with measures of anxiety and depressive symptoms has been investigated. A significant correlation was observed between poor sleep quality and the thickness of the cerebral cortex in the middle portion STS. Furthermore, the thickness of the cerebral cortex in this brain region was found to mediate the association between anxiety, and especially the somatic component of anxiety, as measured by SAS and the physiological and affective components of depression as measured by SDS and poor sleep quality. All of these results remained significant after accounting for age, sex and education level. Furthermore, commonality analyses revealed that the physiological component of SDS accounted for the largest part of the variance in sleep quality. Taken together, these findings suggest that left STS may mediate the influence of anxiety and depressive symptoms, especially the physiological component of depression, on sleep quality.

The observation that poor sleep quality is associated with thinner cortex in the middle portion of left STS is consistent with a previous longitudinal study in which it was reported that poor sleep quality was associated with atrophy of left temporal cortex, including STS (Sexton *et al.*, 2014) and in line with several other studies in which increased cortical atrophy and reduced cortical thickness was reported in people with sleep disturbances (Mander *et al.*, 2013; Dai *et al.*, 2018; Pereira *et al.*, 2019). The size, density and arrangement of neurons, neuroglia and nerve fibers can all influence the thickness of the cerebral cortex (Narr *et al.*, 2005; Geuze *et al.*, 2008), and cortical thinning in STS may be related to decreased neuron number, neuron shrinkage, reduction in synaptic density or changes in myelination (Gogtay *et al.*, 2004; Shaw *et al.*, 2006). A prime function of sleep is to allow repair and restoration of brain function, and sleep has recently been shown to be associated with increased expression of genes involved in the formation and maintenance of myelin (Bellesi *et al.*, 2013). Thus, poor sleep quality may be expected to produce microstructural changes in the brain and have an impact on brain structure, and one possibility is that the directionality of the effect observed in the present study is that poor sleep quality leads to reduced myelination and thinner cerebral cortex especially in left STS. However, it is of course also possible that thinner cortex in left STS is a pre-existing trait in subjects with poor sleep quality. Further longitudinal studies are needed to more fully understand the brain structure–function relationship that has been observed.

There is evidence from functional neuroimaging studies that the middle portion of left STS is involved in processing aspects of human social communication, such as speech (Binder *et al.*, 2000; Jäncke *et al.*, 2002) and vocalization (Belin *et al.*, 2000; Fecteau *et al.*, 2004), integration of auditory and visual sensory information (Balk *et al.*, 2010), and emotion perception, including processing of non-verbal emotional signals and emotional facial expressions (Kreifelts *et al.*, 2009; Robins *et al.*, 2009; Said *et al.*, 2010). People with anxiety and depressive symptoms exhibit deficits in social competence and emotional functioning, including perception and regulation of emotions (Fisher-Beckfield and McFall, 1982; Campbell-Sills *et al.*, 2006; Ehring *et al.*, 2010; Zilverstand *et al.*, 2017), and have been reported to have impairments in recognition of facial expressions of emotion (Fecteau *et al.*, 2004; Surguladze *et al.*, 2004). In particular, people with anxiety symptoms commonly have difficulty in understanding, reacting to and managing their emotional experiences, and it is possible that the level of emotion dysregulation could effectively predict the presence of anxiety disorder or symptoms (Mennin *et al.*, 2005). Similarly, people who are depressed exhibit anomalies in emotional responses to positive and negative stimuli, such as pleasant and sad films, and a lower degree of sadness reactivity is associated with greater psychosocial impairment in depressed individuals (Rottenberg *et al.*, 2002). From the treatment perspective, emotion regulation strategies, such as reappraisal of emotionally evocative stimuli and mindfulness-based stress reduction, have been reported to reduce negative affect in patients with anxiety and depressive symptoms (Koszycki *et al.*, 2007; Ehring *et al.*, 2010; Goldin and Gross, 2010) and in healthy people (Chiesa and Serretti, 2009). Given the posited function of left STS and abovementioned features of emotional symptoms, the results of the present study suggest that reduction in cortical thickness in left STS may be a potential neural substrate underlying emotional dysfunction in people with anxiety and depressive symptoms.

In the present study, cortical thickness of left STS has been observed to serve as a mediator between sleep quality and especially the somatic component of anxiety, and the affective and, especially, the physiological components of depression. There have been several reports that chronic sleep problems are associated with inappropriate physiological arousal, including elevated heart rate and brain activity and increased whole body and global brain metabolism (Nofzinger *et al.*, 2004; Bonnet and Arand, 2010). Attempts have been made to disentangle the potential causality between physiological hyperarousal and sleep problems, and it has been reported that a range of physiological stressors, especially unresolved emotional distress, which serve as a predisposing factor to produce physiological hyperarousal, will increase the likelihood of insomnia (Cano *et al.*, 2008; Wassing *et al.*, 2016). Anxiety and depressive symptoms are both strongly associated with general distress and negative affect (Watson, 2009; Eysenck and Fajkowska, 2018) and marked by changes in the autonomic nervous and endocrine system, and a variety of somatic and physiological symptoms, such as faster heartbeat, sweating and fatigue (Lopez *et al.*, 2018). People with thinner cortex in left STS may experience heightened emotional misperception and dysregulation associated with symptoms of anxiety and depression and more readily accumulate emotional distress leading to physiological hyperarousal and impairment in sleep quality.

The results of the mediation analyses revealed that variations in the thickness of the middle portion of STS can explain the association between sleep quality and three different sub-scores of the anxiety and depression measures. This provides evidence for high concurrent comorbidity of anxiety and depressive symptoms (Sivertsen *et al.*, 2012; Kahn *et al.*, 2013; Cummings *et al.*, 2014; Hertenstein *et al.*, 2019). Nevertheless, anxiety and depressive symptoms are different mental disorders with distinct features (Eysenck and Fajkowska, 2018), and both symptoms are likely to contribute to sleep quality disturbance in different ways. Commonality analysis was therefore used to explore the unique and shared contributions of the three different sub-scores of the anxiety and depression to sleep quality. The physiological component of depression was found to account for the largest portion of the variation in sleep quality and may thus be the main cause of the debilitating effect that emotional distress has on sleep quality. Attention should thus be given to studying the neurobiological mechanisms that might account for physiological symptoms often seen in people who suffer from those problems.

The mediating role in the association of emotional symptoms and poor sleep quality revealed for left STS in the present study may underlie the comorbidity of poor sleep and emotional symptoms in general population (Cheng *et al.*, 2018). Recently, there have been reports that alterations in brain structure and function underlie the association between sleep problems and emotional symptoms in major depressive disorders (Yu *et al.*, 2018; Zhu *et al.*, 2020; Yang *et al.*, 2020). Although there is increasing evidence of comorbidity of sleep problems and emotional symptoms in the general population, few have assessed the differences after removing patients who had at some time been diagnosed with depression (Cheng *et al.*, 2018).

The present study has several limitations. Firstly, the study population comprises healthy participants rather than patients with formally diagnosed emotional disorders. Secondly, self-reported questionnaires were used to measure sleep quality and levels of anxiety and depressive symptoms. The finding of a highly significant association between sleep quality and emotional symptoms is, however, consistent with other studies in patient cohorts. Thirdly, to increase sample size, investigations performed with two different types of head coils were combined. Fourthly, the cross-sectional design makes it difficult to draw firm conclusions regarding the potential cause of the significant relationship between sleep quality and levels of anxiety and depressive symptoms in the adult population studied. Further studies are needed to elucidate the causal relationships between anxiety and depressive symptoms and sleep quality (Pillai and Drake, 2015).

## Conclusion

Cortical thickness of left STS has been identified as a potential neural substrate mediating the link between poor sleep quality and symptoms of anxiety and depression. In addition, the physiological components of depressive symptoms were observed to play a key role in sleep problems. Left STS may be a potential target region for guiding clinical treatment of sleep problems.

## Supplementary Material

nsab012_Supp

## Data Availability

The data that support the findings of this study are available from the corresponding author upon reasonable request.
