# Supplementary material for: Left superior temporal sulcus morphometry mediates the impact of anxiety and depressive symptoms on sleep quality in healthy adults
Source: Soc Cogn Affect Neurosci. 2021 Jan 29;16(5):492–501. doi: 10.1093/scan/nsab012 (PMC8095089; doi:10.1093/scan/nsab012)
Supplement: nsab012_Supp [file nsab012_supp.zip › suppleme.docx]

**Supplementary Materials:**

**Table S1.** Direct and indirect effects of left STS thickness on the association between PSQI and emotional symptoms as measured by SAS and SDS questionnaires.

**Table S2.** Direct and indirect effects of left STS thickness on the association between PSQI and emotional symptoms as measured by SAS and SDS questionnaires with the relevant sleep items excluded.

**Table S3.** Results of commonality analysis using the somatic component of SAS and the affective and physiological components of SDS with the relevant sleep items excluded from both as predictors and PSQI as outcome.

**Figure S1.** Partial correlation analysis of the association between PSQI and emotional symptoms as measured by SAS and SDS questionnaires after controlling for age, sex and education level.

**Figure S2.** Partial correlation analysis of the association between the sleep-linked superior temporal sulcus thickness and emotional symptoms as measured by SAS and SDS questionnaires after controlling for age, sex and education level.

**Figure S3.** Partial correlation analysis of the association between PSQI and emotional symptoms as measured by SAS and SDS questionnaires with the relevant sleep items excluded after controlling for age, sex and education level

**Figure S4.** Partial correlation analysis of the association between the sleep-linked superior temporal sulcus thickness and emotional symptoms as measured by SAS and SDS questionnaires with the sleep-relevant items excluded and controlling for age, sex and education level.

**Figure S5.** Left superior temporal sulcus thickness mediated the effect of SAS, the somatic component of SAS, and the affective and physiological components of SDS on PSQI.

**Figure S6.** Distribution plot of the PSQI, SAS and SDS as well as all sub-scores of them.

Abbreviations: SAS, Self-rating Anxiety Scale; SDS, Self-rating Depression Scale; PSQI, Pittsburgh Sleep Quality Index; STS, Superior Temporal Sulcus.

**Explanatory Analyses**

**1. Partial correlation analyses and mediation analyses after excluding the relevant sleep items in anxiety and depression tests.**

One of the 20 items in the SDS and 2 of the 20 items in SAS are on the topic of sleep quality. Therefore in order to eliminate the potential influence of collinearity between sleep quality and emotional symptoms, we performed a separate analysis in which the relevant sleep items in SAS (item 4) and SDS (item 19 and 20) were excluded. Furthermore, with age, sex and education level as confounding variables, we used partial correlation analysis to examine the correlations between SAS, SDS, as well as each sub-score of SAS and SDS and (i) PSQI and (ii) STS thickness. Finally, if any of the anxiety or depression scores or sub-scores were also significantly correlated with sleep-linked morphology, it was specified as an independent variable or a dependent variable in a subsequent mediation analyses.

**Results of statistical analyses**

After excluding the relevant sleep items in SAS (item 4) and SDS (item 19 and 20), and controlling for age, sex and education level, all correlations with behavioral assessments remained significant (between SAS and SDS, *r* = 0.720, *p* < 0.001; PSQI and SAS, *r* = 0.356; *p* < 0.001; PSQI and SDS, *r* = 0.366, *p* < 0.001; PSQI and all sub-scores of SAS (affective symptoms, *r* = 0.278, *p* = 0.001; somatic symptoms, *r* = 0.380, *p* < 0.001); PSQI and all sub-scores of SDS (affective symptoms, *r* = 0.339, *p* < 0.001; physiological symptoms, *r* = 0.375, *p* < 0.001; and psychological symptoms, *r* = 0.283, *p* < 0.001).

Furthermore, after excluding the relevant sleep items in SAS (item 4) and SDS (item 19 and 20), and controlling for age, sex and education level, the correlations between cortical thickness of left superior temporal sulcus (STS) and behavioral assessments remained significant (between left STS thickness and SAS, *r* = -0.287, *p* < 0.001; left STS thickness and SDS, *r* = -0.169, *p* = 0.048; left STS thickness and all sub-scores of SAS (affective symptoms, *r* = -0.219, *p* = 0.010; somatic symptoms, *r* = -0.262, *p* = 0.002), left STS thickness and affective and physiological symptoms of SDS (*r* = -0.205, *p* = 0.016; *r* = -0.218, *p* = 0.010, respectively)).

In addition, after excluding the relevant sleep items in SAS (item 4) and SDS (item 19 and 20), and controlling for age, sex and education level, all mediation models were still maintained. The results showed the left STS thickness significantly mediated the effect of SAS (β = 0.037, SE = 0.019, *p* < 0.05), and the somatic symptoms of SAS (β = 0.050, SE = 0.015, *p* < 0.05), and the affective and physiological symptoms of SDS (β = 0.161, SE = 0.072, *p* < 0.05; β = 0.058, SE = 0.025, *p* < 0.05, respectively) on PSQI and with age, sex and education level accounted for as confounding variables. In the reverse direction, the mediating effect of the left STS thickness on the effect of PSQI on SAS, SDS or corresponding sub-scores was not significant.

**2. Commonality analysis**

In order to determine the unique and shared variance in the contributions of the somatic component of SAS, and the physiological and affective components of SDS, on sleep quality, a commonality analysis was conducted using yhat package in R-studio (https://www.rdocumentation.org/packages/yhat/versions/2.0-0/topics/yhat-package). The commonality analysis decomposes the associations into their unique and shared variance components（Newton & Spurrell, 1967）and allows quantification of how much variance in common and accounted for by two or more predictors (i.e., overlap) (Nathans, Oswald, & Nimon, 2012). A bootstrapping analysis was also performed (1,000 bootstraps) to determine the precision of the estimates and to examine whether certain variables contributed more unique variance in each dependent variable than other independent variables. The estimate can be interpreted in terms of effect sizes (e.g., < 1% negligible, > 1% small, > 9% moderate, and > 25% large) (Cohn 1988) and inspection can be made of whether the confidence intervals from the bootstrapping extend into the negligible range.

**Results of commonality analysis**

Overall, the combination of the somatic component sub-score of SAS and affective and physiological component sub-scores of SDS explained 22.33% of the variance in PSQI. Next highest was the physiological component sub-score of SDS which could explain 18.01% of the variance and the combination of the somatic component sub-score of SAS and physiological component sub-score of SDS could explain 4.5% of the variance, respectively. The physiological component sub-score of SDS could explain an additional 5.91% of the variation in PSQI. In contrast, the somatic component sub-score of SAS, affective component sub-score of SDS and the combination of the latter two sub-scores could explain only 1.4%, 2.22% and 0.7% of the variation in PSQI, respectively.

**Table S1.** Direct and indirect effects of left STS thickness on the association between PSQI and emotional symptoms as measured by SAS and SDS questionnaires.

| Pathway | β | SE(B) | t | p-value | 95%CI |
| --- | --- | --- | --- | --- | --- |
| Anxiety |  |  |  |  |  |
| a | -0.042 | 0.012 | -3.500 | 0.001 | [-0.065, -0.018] |
| b | -0.731 | 0.177 | -4.138 | <0.001 | [-1.080, -.381] |
| c | 0.167 | 0.041 | 4.104 | <0.001 | [0.087, 0.248] |
| c’ | 0.137 | 0.039 | 3.526 | 0.001 | [0.060, 0.214] |
| c-c’ | 0.030 | 0.012 | - | - | [0.012, 0.058] * |
| Somatic component of anxiety |  |  |  |  |  |
| a | -0.059 | 0.015 | -3.872 | <0.001 | [-0.090, -0.029] |
| b | -0.698 | 0.184 | -3.782 | <0.001 | [-1.063, -0.333] |
| c | 0.224 | 0.068 | 3.289 | 0.001 | [0.089, 0.359] |
| c’ | 0.183 | 0.066 | 2.754 | 0.007 | [0.051, 0.314] |
| c-c’ | 0.041 | 0.015 | - | - | [0.018, 0.077]* |
| Affective component of depression |  |  |  |  |  |
| a | -0.194 | 0.075 | -2.573 | 0.011 | [-0.343, -0.045] |
| b | -0.831 | 0.207 | -4.017 | <0.001 | [-1.239, -0.422] |
| c | 0.714 | 0.173 | 4.133 | <0.001 | [0.372, 1.056] |
| c’ | 0.553 | 0.173 | 3.187 | 0.002 | [0.210, 0.896] |
| c-c’ | 0.161 | 0.072 | - | - | [0.047, 0.345]* |
| Physiological component of depression |  |  |  |  |  |
| a | -0.080 | 0.025 | -3.240 | 0.002 | [-0.129, -0.031] |
| b | -0.651 | 0.171 | -3.811 | <0.001 | [-0.988, -0.313] |
| c | 0.382 | 0.066 | 5.796 | <0.001 | [0.252, 0.512] |
| c’ | 0.330 | 0.062 | 5.292 | <0.001 | [0.207, 0.453] |
| c-c’ | 0.052 | 0.020 | - | - | [0.020, 0.099]* |

Note: Pathway a: the effect of emotional symptoms on the mediator (STS); Pathway b: the effect of the mediator (STS) on the outcome (sleep quality); Pathway c: the total effect of the emotional symptoms on the outcome (sleep quality); Pathway c’: the direct effect of the emotional symptoms on the outcome (sleep quality) controlling for the mediator (STS). * With 10,000 bias-corrected bootstrap samples.

**Table S2.** Direct and indirect effects of left STS thickness on the association between PSQI and emotional symptoms as measured by SAS and SDS questionnaires with the relevant sleep items excluded.

| Pathway | β | SE(B) | t | p-value | 95%CI |
| --- | --- | --- | --- | --- | --- |
| Anxiety |  |  |  |  |  |
| a | -0.047 | 0.014 | -3.353 | 0.001 | [-0.075, -0.019] |
| b | -0.790 | 0.194 | -4.068 | <0.001 | [-1.174, -0.406] |
| c | 0.151 | 0.040 | 3.789 | <0.001 | [0.072, 0.230] |
| c’ | 0.114 | 0.038 | 2.992 | 0.003 | [0.039, 0.189] |
| c-c’ | 0.037 | 0.015 | - | - | [0.015, 0.074]* |
| Somatic component of anxiety |  |  |  |  |  |
| a | -0.061 | 0.019 | -3.281 | 0.001 | [-0.098, -0.024] |
| b | -0.816 | 0.197 | -4.139 | <0.001 | [-1.206, -0.426] |
| c | 0.192 | 0.068 | 2.804 | 0.006 | [0.057, 0.327] |
| c’ | 0.142 | 0.065 | 2.190 | 0.030 | [0.014, 0.270] |
| c-c’ | 0.050 | 0.019 | - | - | [0.019, 0.098]* |
| Affective component of depression |  |  |  |  |  |
| a | -0.194 | 0.075 | -2.573 | 0.011 | [-0.343, -0.045] |
| b | -0.831 | 0.207 | -4.017 | <0.001 | [-1.239, -0.422] |
| c | 0.714 | 0.173 | 4.133 | <0.001 | [0.372, 1.056] |
| c’ | 0.553 | 0.173 | 3.187 | 0.002 | [0.210, 0.896] |
| c-c’ | 0.161 | 0.072 | - | - | [0.047, 0.345]* |
| Physiological component of depression |  |  |  |  |  |
| a | -0.075 | 0.028 | -2.720 | 0.007 | [-0.130, -0.021] |
| b | -0.765 | 0.179 | -4.275 | <0.001 | [-1.119, -0.411] |
| c | 0.347 | 0.070 | 4.947 | <0.001 | [0.208, 0.486] |
| c’ | 0.290 | 0.064 | 4.518 | <0.001 | [0.163, 0.416] |
| c-c’ | 0.058 | 0.025 | - | - | [0.017, 0.115]* |

Note: Pathway a: the effect of emotional symptoms on the mediator (STS); Pathway b: the effect of the mediator (STS) on the outcome (sleep quality); Pathway c: the total effect of the emotional symptoms on the outcome (sleep quality); Pathway c’: the direct effect of the emotional symptoms on the outcome (sleep quality) controlling for the mediator (STS). * With 10,000 bias-corrected bootstrap samples.

**Table S3.** Results of commonality analysis using the somatic component of SAS and the affective and physiological components of SDS with the relevant sleep items excluded from both as predictors and PSQI as outcome.

| Variable | Variance (%) | 95% bootstrap confidence intervals (CI) | Model R2 |
| --- | --- | --- | --- |
| SDS_Physiological (U1) | 5.91 | [0.015, 0.134] | 0.180 |
| SAS_Somatic (U2) | 1.40 | [0.000, 0.092] | 0.112 |
| SDS_Affective (U3) | 2.22 | [0.000, 0.079] | 0.105 |
| SDS_Physiological, SAS_Somatic (C1) | 4.55 | [0.004, 0.104] | 0.201 |
| SDS_Physiological, SDS_Affective (C2) | 2.90 | [0.003, 0.075] | 0.209 |
| SDS_Affective, SAS_Somatic (C3) | 0.70 | [-0.002, 0.030] | 0.164 |
| SDS_Physiological, SDS_Affective, SAS_Somatic (C4) | 4.66 | [0.014, 0.092] | 0.223 |

U1 and U2 and U3: variance in PSQI explained uniquely (i.e., specificity) by physiological component of SDS (U1), somatic component of SAS (U2) and affective component of SDS (U3), respectively. C1: variance in PSQI explained by the overlap between physiological component of SDS and somatic component of SAS. C2: variance in PSQI explained by the overlap between physiological and affective components of SDS. C3: variance in PSQI explained by the overlap between somatic component of SAS and affective component of SDS. C4: variance in PSQI explained by the combination of the somatic component of SAS and affective and physiological components of SDS.

**Figure S1.** Partial correlation analysis of the association between PSQI and emotional symptoms as measured by SAS and SDS questionnaires after controlling for age, sex and education level.


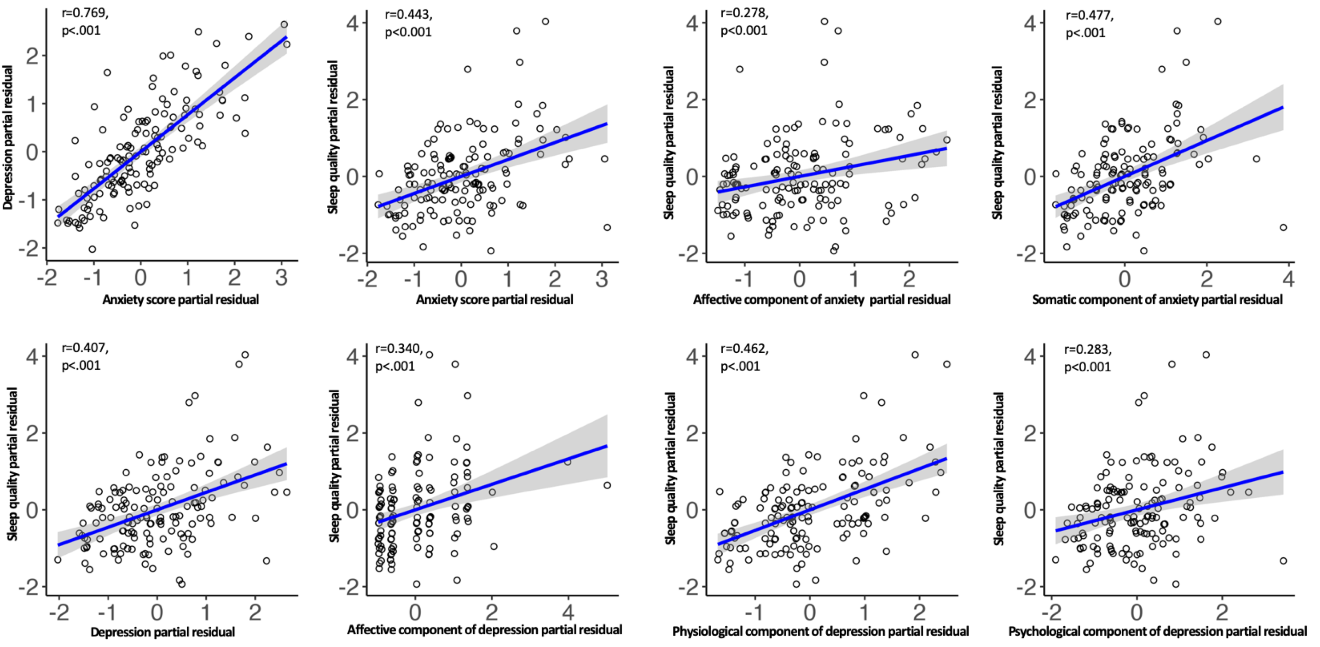


**Figure S2.** Partial correlation analysis of the association between the sleep-linked superior temporal sulcus thickness and emotional symptoms as measured by SAS and SDS questionnaires after controlling for age, sex and education level.


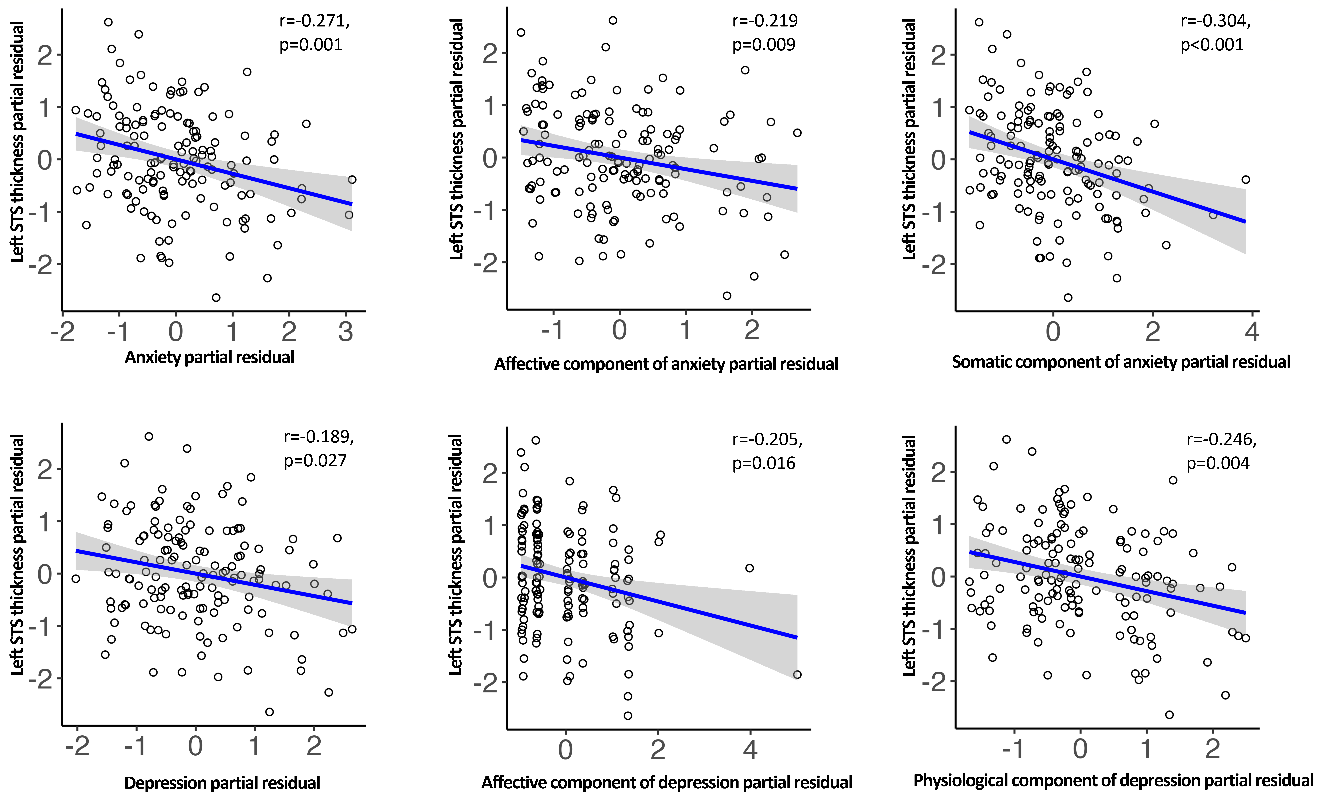


**Figure S3.** Partial correlation analysis of the association between PSQI and emotional symptoms as measured by SAS and SDS questionnaires with the relevant sleep items excluded by partial correlation analyses and controlling for age, sex and education level.


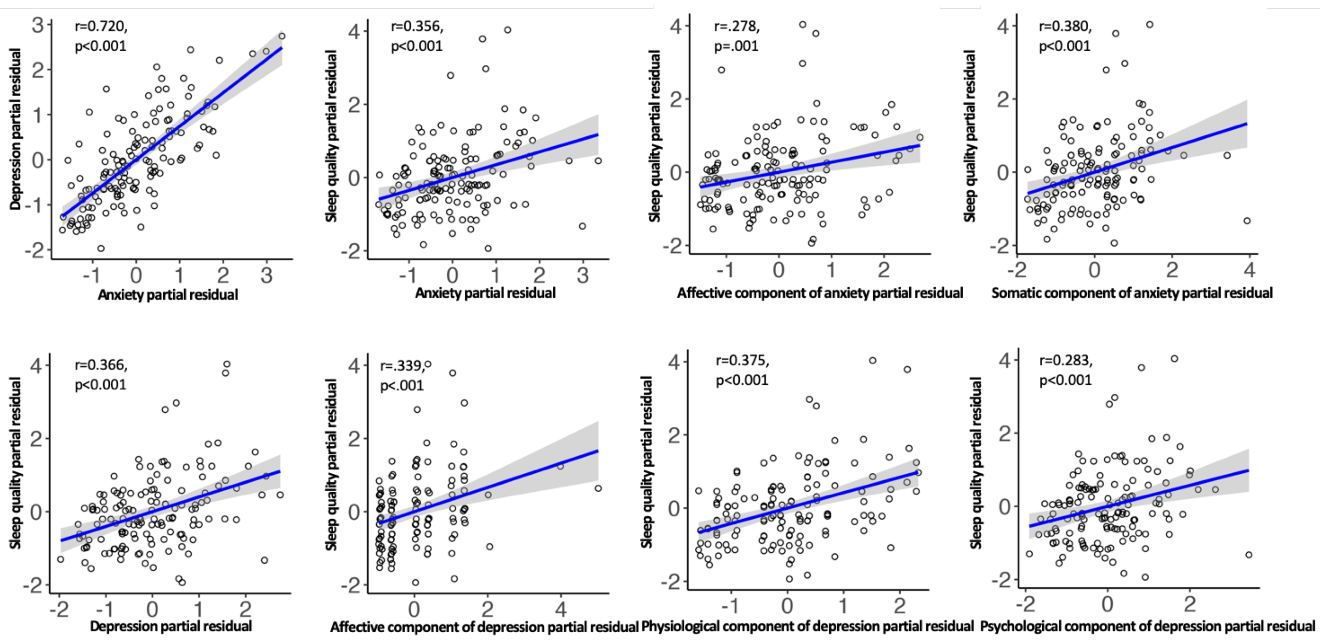


**Figure S4.** Partial correlation analysis of the association between the sleep-linked superior temporal sulcus thickness and emotional symptoms as measured by SAS and SDS questionnaires with the sleep-relevant items excluded and controlling for age, sex and education level.


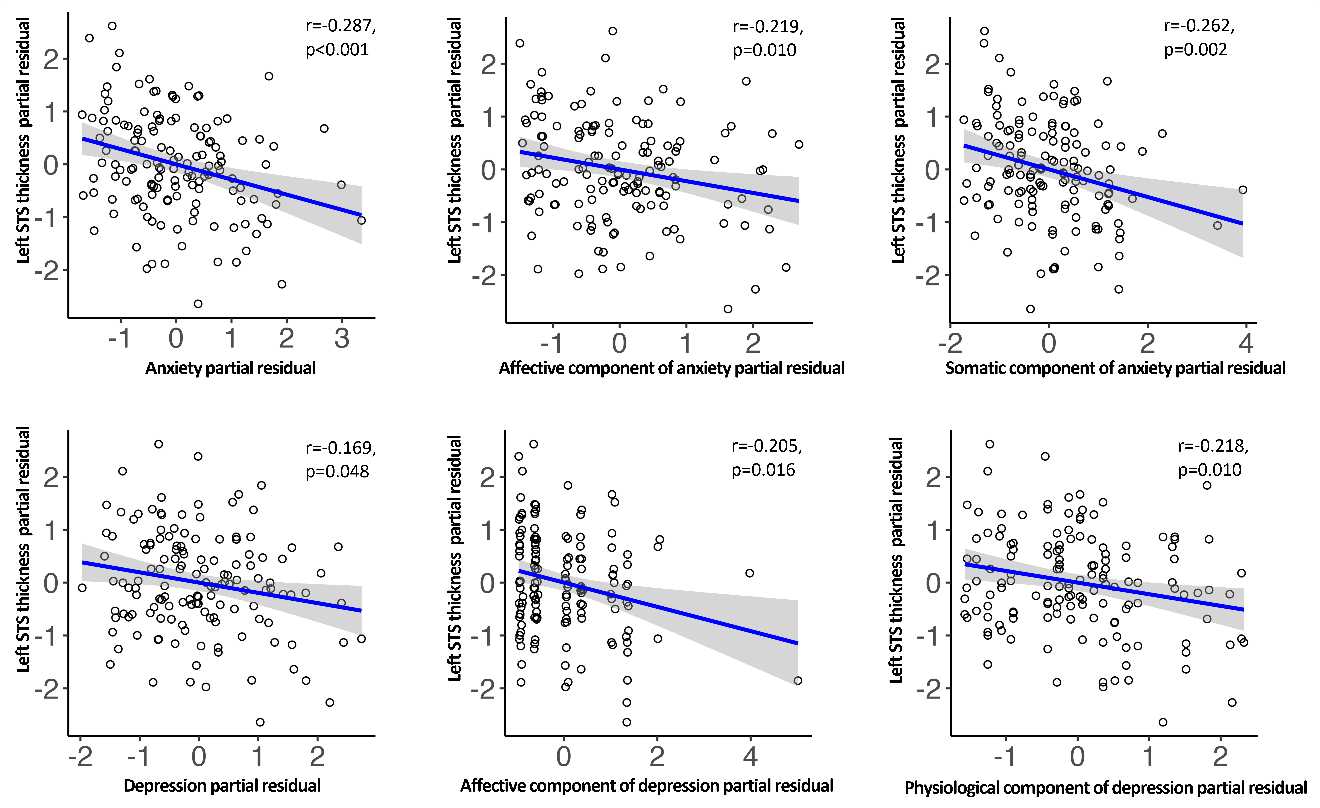


**Figure S5.** Left superior temporal sulcus thickness mediated the effect of SAS, the somatic component of SAS, and the affective and physiological components of SDS on PSQI. The relevant sleep items in SAS and SDS were excluded in the mediation analyses.


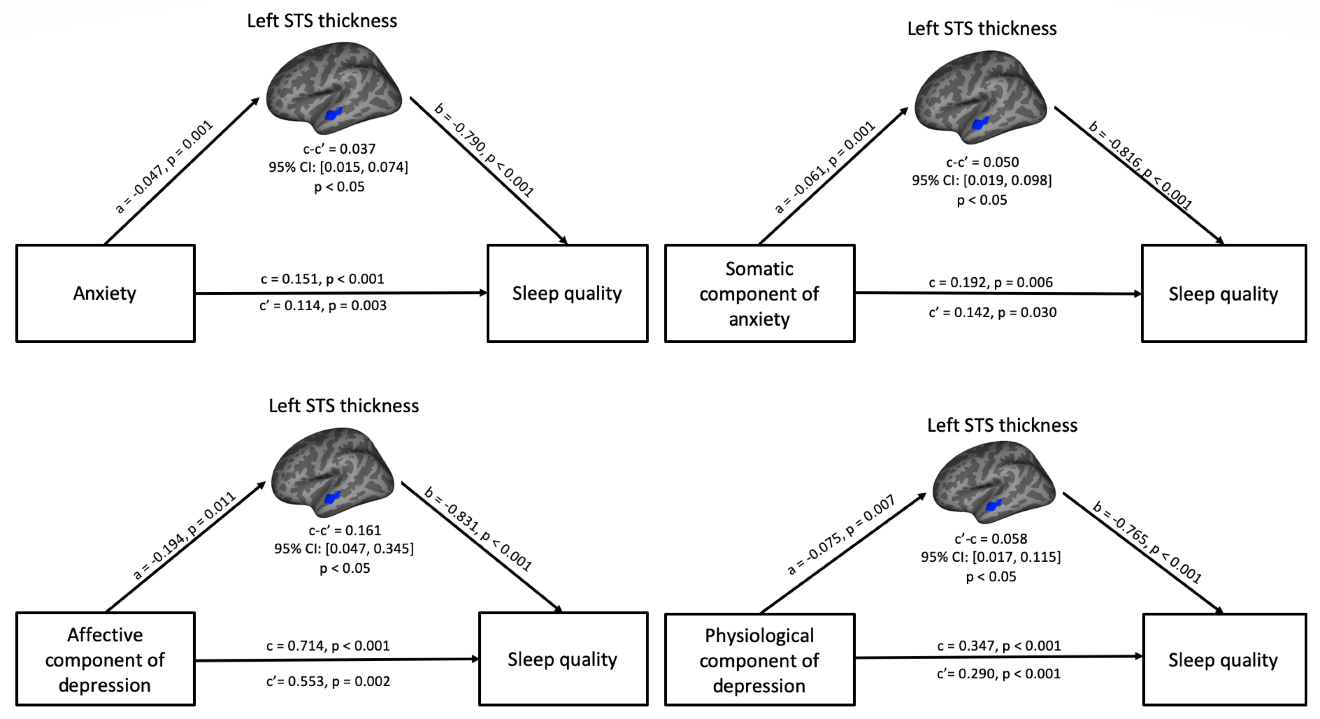


**Figure S6.** Distribution plot of the PSQI, SAS and SDS as well as the subscores of them. Note: the red dashed line represents mean value.


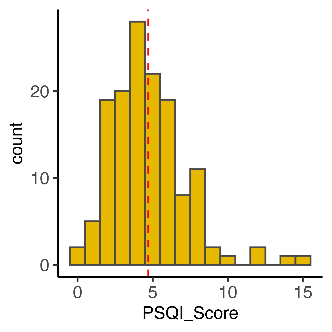

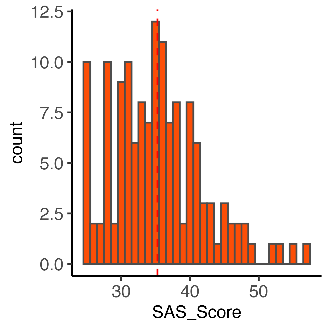

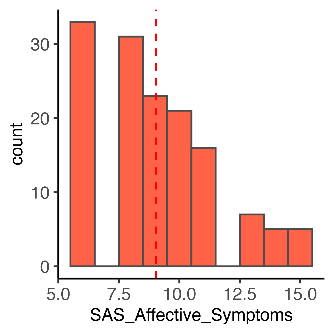

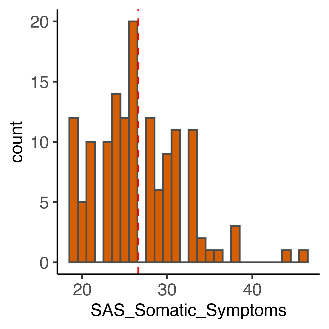


**
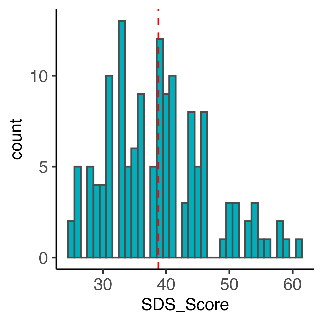

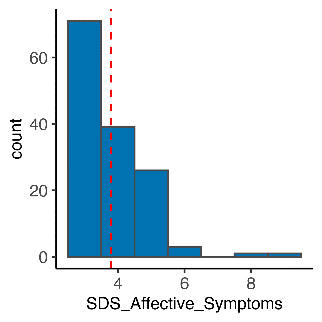

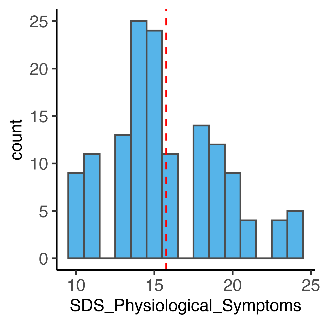

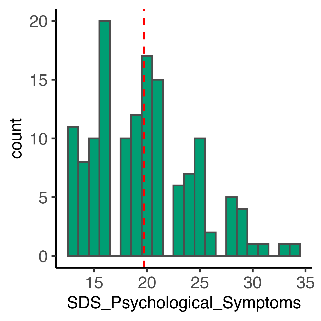
**

**References**

Cohn J. (1988). Statistical power analysis for the behavioral sciences. Lawrence Earlbam Associates, Hillsdale, NJ.

Nathans LL, Oswald FL, Nimon K. (2012). Interpreting multiple linear regression: A guidebook of variable importance. Practical Assessment, Research, and Evaluation.,17: 1-19.

Newton R, Spurrell D. (1967). A development of multiple regression for the analysis of routine data. Journal of the Royal Statistical Society: Series C (Applied Statistics), 16: 51-64.
